# Focus on materials challenges for protection - environment and health

**DOI:** 10.1088/1468-6996/16/3/030301

**Published:** 2015-05-14

**Authors:** Harald Krug

**Affiliations:** Swiss Federal Laboratories for Materials Science and Technology (Empa), Switzerland

Environment, human health and safety (EHS) are ever more important topics for new applications of advanced and so-called ‘smart’ materials. Today our daily life is dependent on innovative material developments and new systems for sustainable applications, and this dependence will only increase in the future. Alternative energy production and storage; water, soil and air remediation; and the use of new materials on and in the human body for medical purposes or body function recovery are just a few examples. A new impulse for EHS aspects is brought about by the advent of nanotechnology. Highly active catalysts, improved energy storage systems, solar cells with enhanced efficiency, water filters and waste treatment systems can be build up with increasingly smaller units based on nanomaterials. Moreover, very efficient drug delivery systems and early diagnostics will be possible in the near future owing to nanotechnological developments.

We probably enter into a new era of environmental challenges. The larger temperature differences between warm and cold regions on our planet will most likely increase the number and speed of thunderstorms like the cyclone ‘Pam’ and the hurricane ‘Katrina’. To reduce their likelihood we should refrain from burning oil-based fuels. We have to develop more sustainable approaches such as the use of alternative energy sources. Our building environment should be adapted as well and this holds true for streets, tunnels and slope protection. Here, insulation with nanostructured materials, new polymers and ‘geotextiles’ offers new possibilities. In the near future materials will sense failures as well as stimuli from the environment and respond to them accordingly.

Sensing is also a big issue for human health. Hydrogels or polymers will monitor their surrounding tissue and, for instance, release drugs or attract cells for advanced wound healing. Therapies and healing processes may be supported by material implants responding to light, temperature or other physical and chemical stimuli without a direct contact. Medical applications will change profoundly in the near future; targeted drugs, better treatments through the skin and the lungs and smart systems supporting body functions will reduce side effects and enhance therapeutic success.

Besides all these developments we should also consider the other side of the medal, the possible risks of such new systems and materials. The discussion started already years ago about possible EHS-related effects especially with respect to nanomaterials. Although we know a lot about safety issues of nanomaterials, the past 15 years clearly showed that we have deficits in specific knowledge and the biological safety testing of such new materials. That is why we will establish a new platform for nanomaterials testing and will publish with high priority the so-called ‘no-effect studies’. Such information is rarely published as journals usually accept only studies, which show an effect. Thus, the information is unbalanced with respect to the possible hazards of nanomaterials and the subsequent risk assessment. We want to help the scientific community with this platform to get back on track for a sustainable risk management.

New developments of materials for applications in the environment and health fields require intensive investigations for both material design and material safety—topics of significant interest in this focus issue.

I would like to thank all authors who contributed to this focus issue, and I do hope that we will have a balanced discussion about benefits and risks of new materials in the future.

